# Evidence for species-dependent biosynthetic pathways for converting carlactone to strigolactones in plants

**DOI:** 10.1093/jxb/erx428

**Published:** 2017-12-23

**Authors:** Moe Iseki, Kasumi Shida, Kazuma Kuwabara, Takatoshi Wakabayashi, Masaharu Mizutani, Hirosato Takikawa, Yukihiro Sugimoto

**Affiliations:** Graduate School of Agricultural Science, Kobe University, Kobe, Japan

**Keywords:** Biosynthesis, carlactone, carlactonoic acid, 4-deoxyorobanchol, 5-deoxystrigol, strigolactone

## Abstract

Strigolactones (SLs), comprising compounds with diverse but related chemical structures, are determinant signals in elicitation of germination in root parasitic Orobanchaceae and in mycorrhization in plants. Further, SLs are a novel class of plant hormones that regulate root and shoot architecture. Dissecting common and divergent biosynthetic pathways of SLs may provide avenues for modulating their production *in planta*. Biosynthesis of SLs in various SL-producing plant species was inhibited by fluridone, a phytoene desaturase inhibitor. The plausible biosynthetic precursors of SLs were exogenously applied to plants, and their conversion to canonical and non-canonical SLs was analysed using liquid chromatography–tandem mass spectrometry. The conversion of carlactone (CL) to carlactonoic acid (CLA) was a common reaction in all investigated plants. Sorghum converted CLA to 5-deoxystrigol (5-DS) and sorgomol, and 5-DS to sorgomol. One sorgomol-producing cotton cultivar had the same SL profile as sorghum in the feeding experiments. Another cotton cultivar converted CLA to 5-DS, strigol, and strigyl acetate. Further, 5-DS was converted to strigol and strigyl acetate. Moonseed converted CLA to strigol, but not to 5-DS. The plant did not convert 5-DS to strigol, suggesting that 5-DS is not a precursor of strigol in moonseed. Similarly, 4-deoxyorobanchol was not a precursor of orobanchol in cowpea. Further, sunflower converted CLA to methyl carlactonoate and heliolactone. These results indicated that the biosynthetic pathways of hydroxy SLs do not necessarily involve their respective deoxy SL precursors.

## Teaser

Strigolactones, a novel class of phytohormones, regulate plant architecture and act as rhizosphere signals. Species-specific biosynthetic pathways convert a common precursor, carlactone, to strigolactones.

## Introduction

**Fig. 1.**
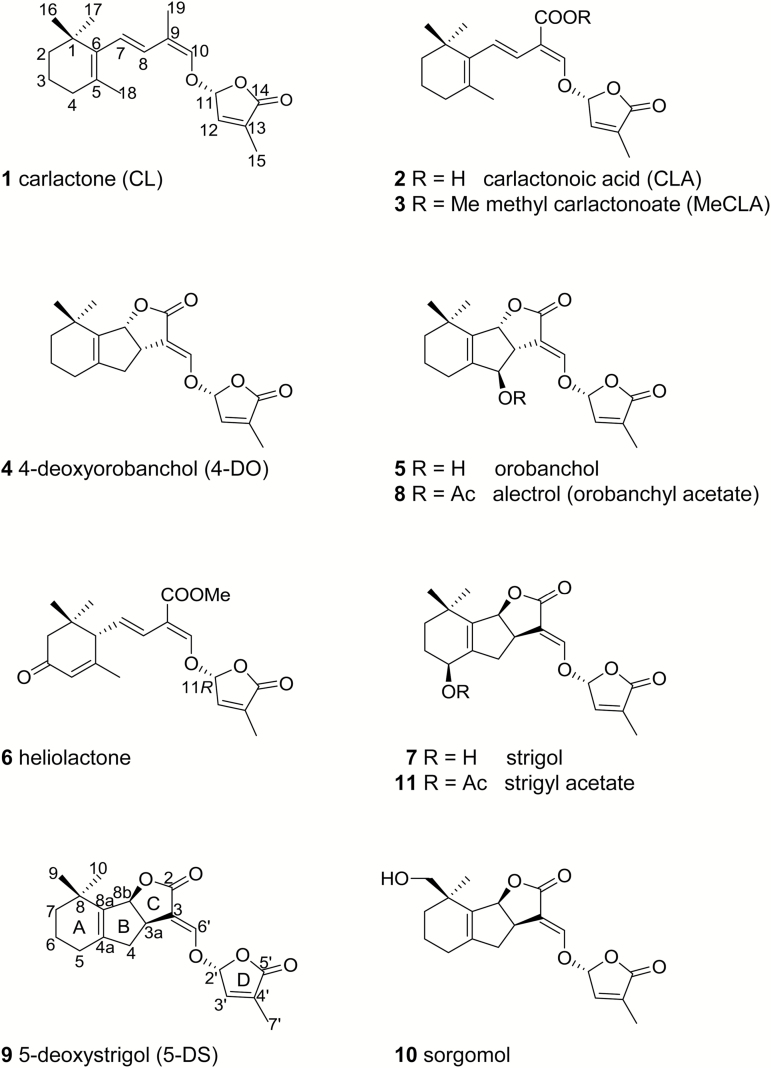
Structures of naturally occurring canonical and non-canonical strigolactones.

**Fig. 2.**
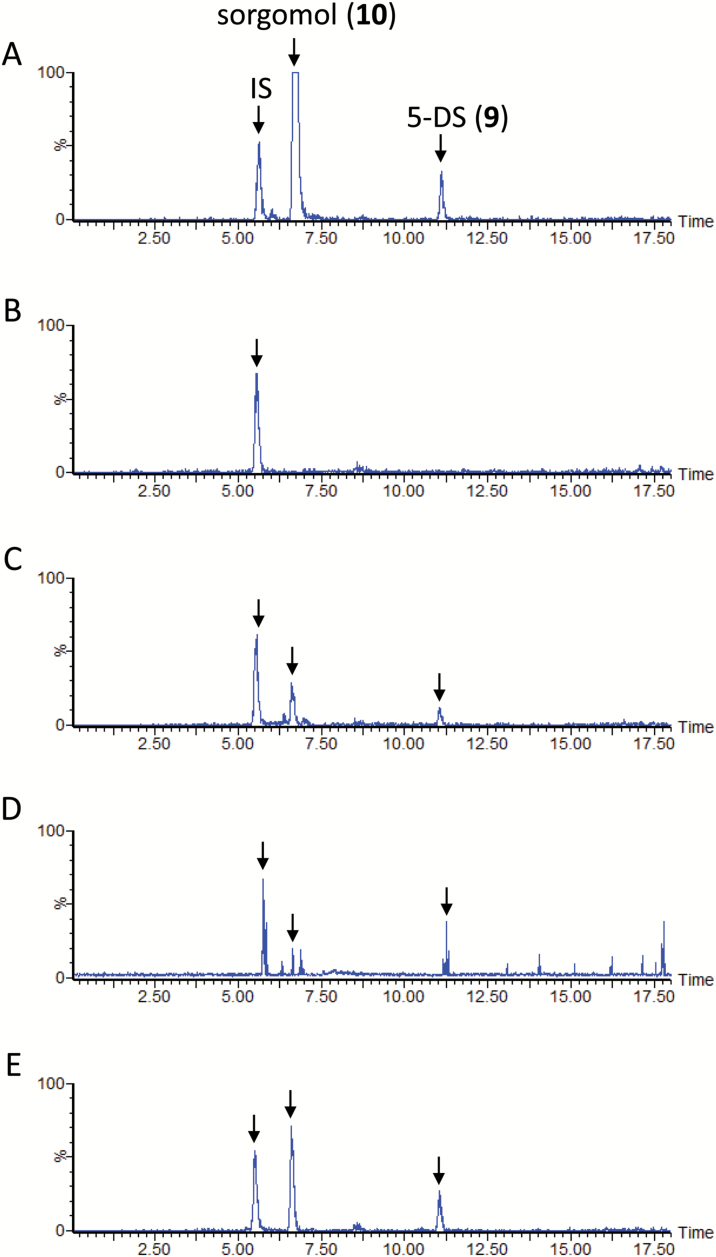
Liquid chromatography–tandem mass spectrometry analysis of sorghum root exudates. The total ion current chromatograms were integrations of multiple reaction monitoring chromatograms with transitions selected for strigol, sorgomol, 5-deoxystrigol (5-DS), and carlactone (CL). (A) Aquaculture filtrate of sorghum grown under phosphate-deficient conditions. (B) Aquaculture filtrate of sorghum grown under phosphate-deficient conditions and supplemented with fluridone. (C) Conversion of CL (**1**) to 5-DS (**9**) and sorgomol (**10**). (D) Conversion of carlactonoic acid (CLA) (**2**) to 5-DS (**9**) and sorgomol (**10**). (E) Conversion of 5-DS (**9**) to sorgomol (**10**). IS, internal standard.

**Fig. 3.**
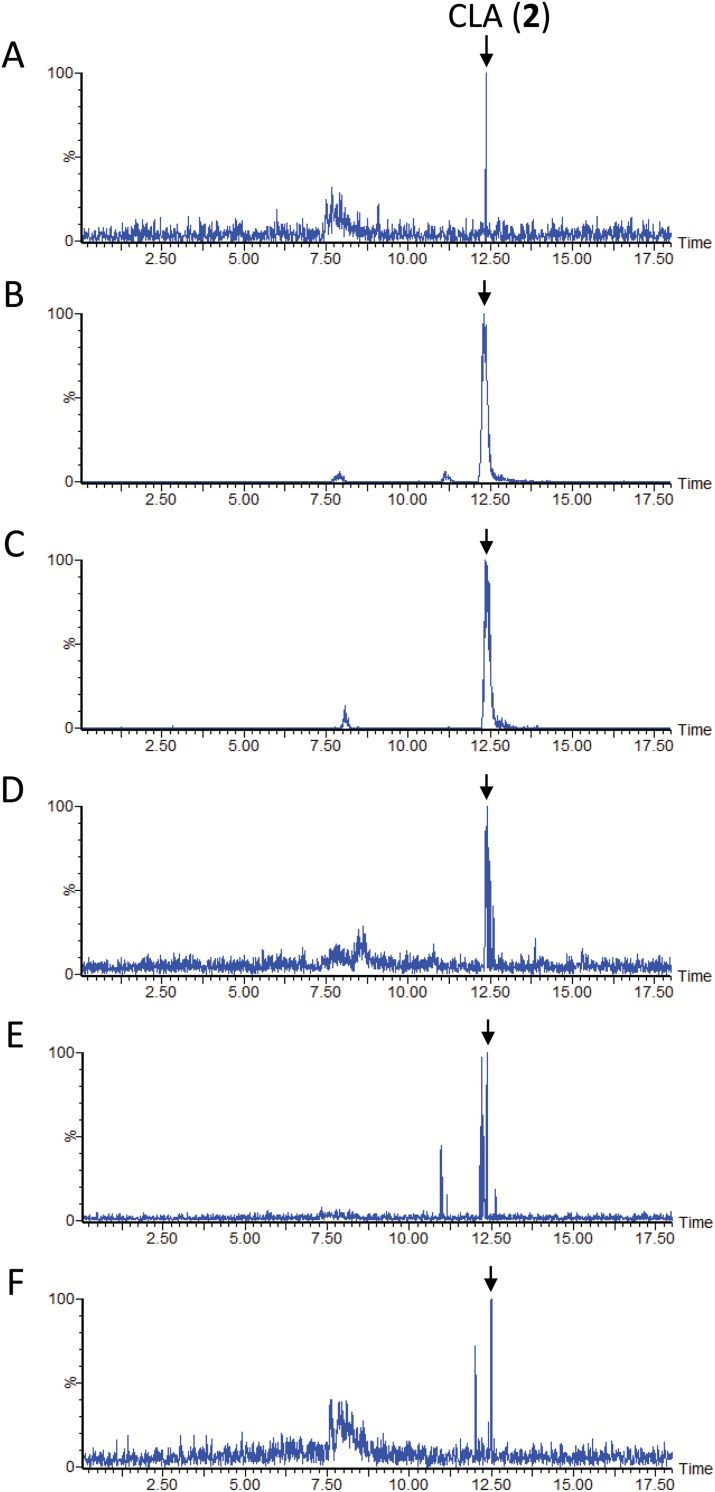
Liquid chromatography–tandem mass spectrometry analysis of carlactonoic acid (CLA) (**2**) in aquaculture filtrate of several plants fed with carlactone (CL) (**1**). Multiple reaction monitoring channel was at *m*/*z* 331.2>113.0. (A) Sorghum; (B) Wawata cotton; (C) Tonko cotton; (D) moonseed; (E) cowpea; (F) sunflower. Chromatogram signal intensities for CLA were 1.45 × 10^3^ (A), 4.31 × 10^5^ (B), 1.53 × 10^5^ (C), 2.41 × 10^3^ (D), 5.26 × 10^3^ (E), and 1.82 × 10^3^ (F).

**Fig. 4.**
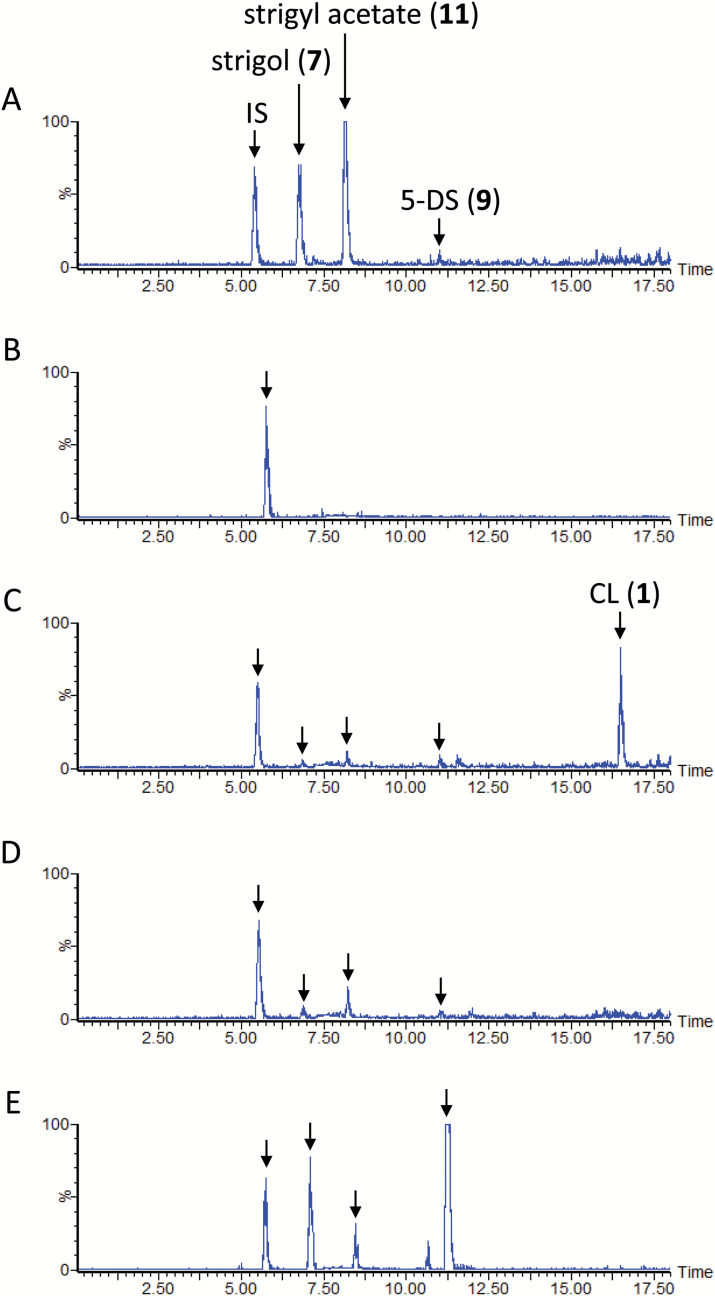
Liquid chromatography–tandem mass spectrometry analysis of Tonko cotton root exudates. The total ion current chromatograms were integrations of multiple reaction monitoring chromatograms with transitions selected for strigol, strigyl acetate, sorgomol, 5-deoxystrigol (5-DS), and carlactone (CL). (A) Aquaculture filtrate of cotton grown under phosphate-deficient conditions. (B) Aquaculture filtrate of cotton grown under phosphate-deficient conditions and supplemented with fluridone. (C) Conversion of CL (**1**) to 5-DS (**9**), strigol (**7**), and strigyl acetate (**11**). (D) Conversion of carlactonoic acid (CLA) (**2**) to 5-DS (**9**), strigol (**7**), and strigyl acetate (**11**). (E) Conversion of 5-DS (**9**) to strigol (**7**) and strigyl acetate (**11**). IS, internal standard.

**Fig. 5.**
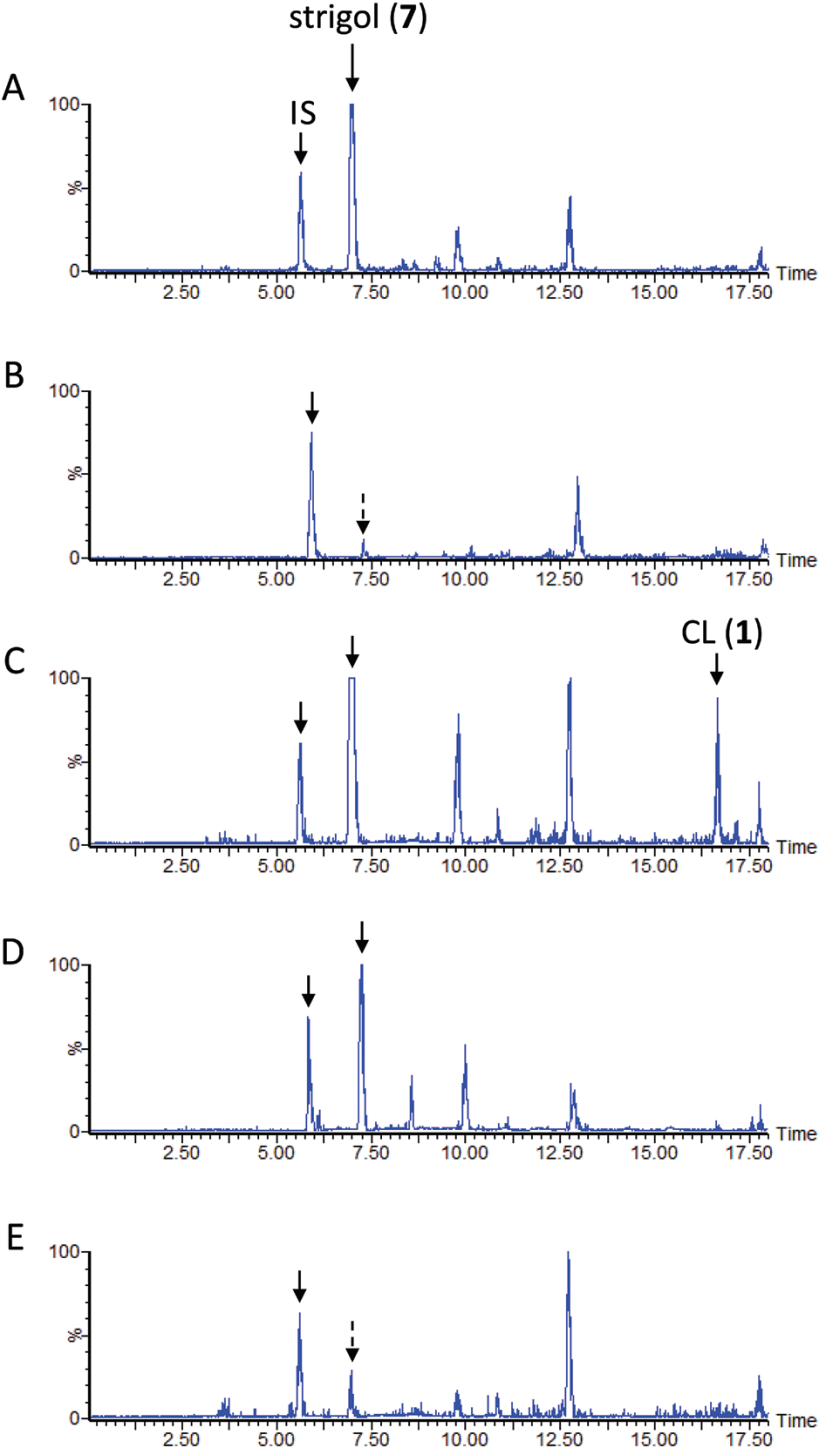
Liquid chromatography–tandem mass spectrometry analysis of moonseed root exudates. The total ion current chromatograms were integrations of multiple reaction monitoring chromatograms with transitions selected for strigol, strigyl acetate, 5-deoxystrigol (5-DS), and carlactone (CL). (A) Root culture filtrate of moonseed grown under phosphate-deficient conditions. (B) Root culture filtrate of moonseed grown under phosphate-deficient conditions and supplemented with fluridone. (C) Conversion of CL (**1**) to strigol (**7**). (D) Conversion of carlactonoic acid (CLA) (**2**) to strigol (**7**). (E) 5-DS (**9**) was not converted to strigol (**7**). IS, internal standard.

**Fig. 6.**
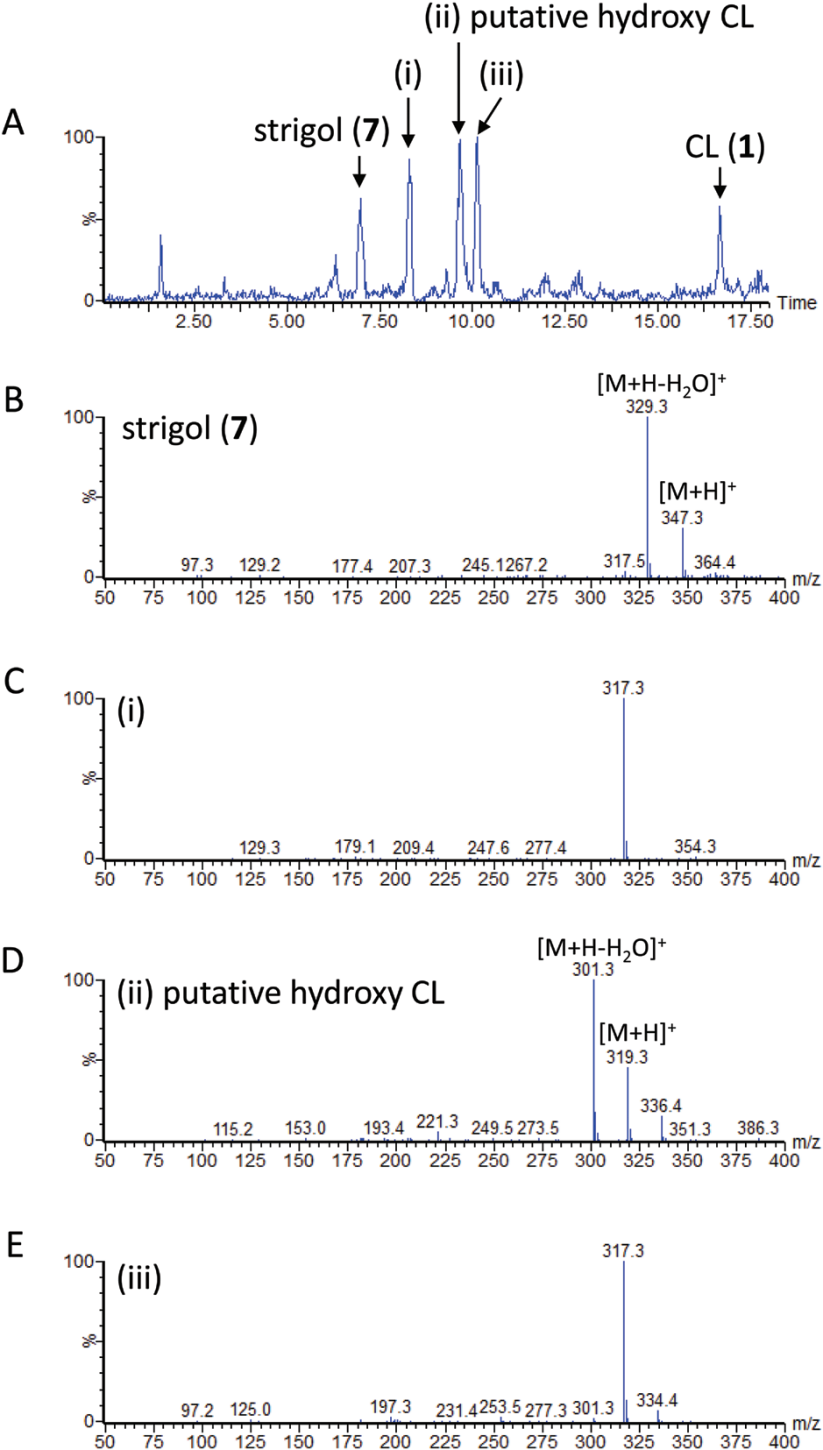
Liquid chromatography–tandem mass spectrometry analysis of moonseed root exudates. Parent ion scan was performed for the fragment ion at *m*/*z* 97. (A) Root culture filtrate of moonseed grown under phosphate-deficient conditions supplemented with fluridone and a larger amount of carlactone (CL) (**1**). Mass spectra of strigol (B) and the unidentified CL metabolites eluted at 8.3 min (C), 9.7 min (D), and 10.1 min (E).

Strigolactones (SLs) were first isolated from cotton root exudates and identified as germination stimulants of witchweed seeds (Cook *et al.*, 1966). Since the beginning of this century, significant physiological functions of SLs have been revealed, including the induction of the symbiotic association between plant roots and mycorrhizal fungi (Akiyama *et al.*, 2005), inhibition of bud outgrowth to decrease shoot branching (Gomez-Roldan *et al.*, 2008; Umehara *et al.*, 2008), and regulation of root architecture (Brewer *et al.*, 2013). These findings provided a rational explanation for the long unresolved question of why host plants produce and secrete SLs that trigger the germination of root parasitic weeds in their rhizosphere. Typically, SLs consist of a tricyclic lactone (ABC-ring) connected to a methylbutenolide (D-ring) via an enol ether bridge. These structures are collectively designated as canonical SLs. Considering their physiological significance in plants, the biosynthesis, perception, and signal transduction of SLs have attracted increasing attention. Matusova *et al.* (2005), using abscisic acid (ABA)-deficient maize mutants, showed that SLs are derived from a carotenoid precursor. D27, a *cis*–*trans* isomerase, catalyses the conversion of β-carotene to 9-*cis*-β-carotene, which is cleaved by CCD7 to yield 9-*cis*-β-apo-10ʹ-carotenal. The aldehyde is cleaved by CCD8, and the product then undergoes an intramolecular transformation to form carlactone (CL; Fig. 1, **1**; Alder *et al.*, 2012). In Arabidopsis, MAX1 (CYP711A1) catalyses the conversion of CL (**1**) to carlactonoic acid (CLA; Fig. 1, **2**; Abe *et al.*, 2014), which is methylated to methyl carlactonoate (MeCLA; Fig. 1, **3**), and then oxidized by lateral branching oxidoreductase to an unidentified SL-like compound with molecular mass larger than that of MeCLA (**3**) by 16 Da (Brewer *et al.*, 2016). Two CYP711A enzymes catalyse two distinct steps in rice: BC-ring closure of CL (**1**) by carlactone oxidase (Os01g0700900) to yield 4-deoxyorobanchol (4-DO; Fig. 1, **4**) and a subsequent structural diversification step to produce orobanchol (Fig. 1, **5**) via orobanchol synthase (Os01g0701400) (Zhang *et al.*, 2014; Al-Babili and Bouwmeester, 2015). The SLs in which the BC ring is unclosed are designated as non-canonical. These include CL (**1**), CLA (**2**), MeCLA (**3**), and heliolactone (Fig. 1, **6**) (Ueno *et al.*, 2014). An α/β hydrolase (D14 or DAD2), an F-box protein (MAX2, D3, or RMS4), and a protein (D53 or SMXL6/7/8) are identified as the components required for SL perception and signal transduction (Khosla and Nelson, 2016).

**Fig. 7.**
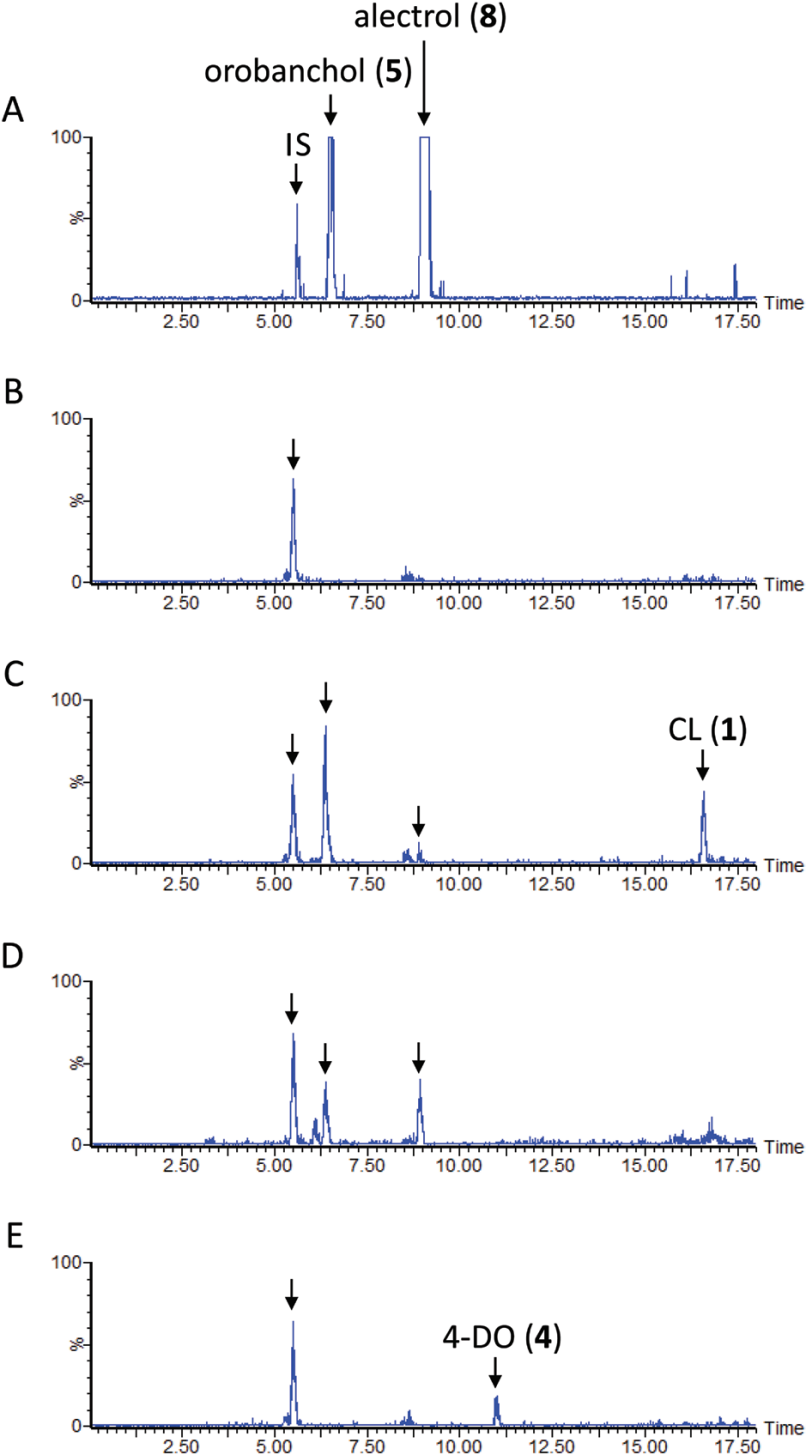
Liquid chromatography–tandem mass spectrometry analysis of cowpea root exudates. The total ion current chromatograms were integrations of multiple reaction monitoring chromatograms with transitions selected for strigol, orobanchol, alectrol, 5-deoxystrigol (5-DS), 4-deoxyorobanchol (4-DO), and carlactone (CL). (A) Aquaculture filtrate of cowpea grown under phosphate-deficient conditions. (B) Aquaculture filtrate of cowpea grown under phosphate-deficient conditions and supplemented with fluridone. (C) Conversion of CL (**1**) to orobanchol (**5**) and alectrol (**8**). (D) Conversion of carlactonoic acid (CLA) (**2**) to orobanchol (**5**) and alectrol (**8**). (E) 4-DO (**4**) was not converted to orobanchol (**6**) or alectrol (**8**). IS, internal standard.

**Fig. 8.**
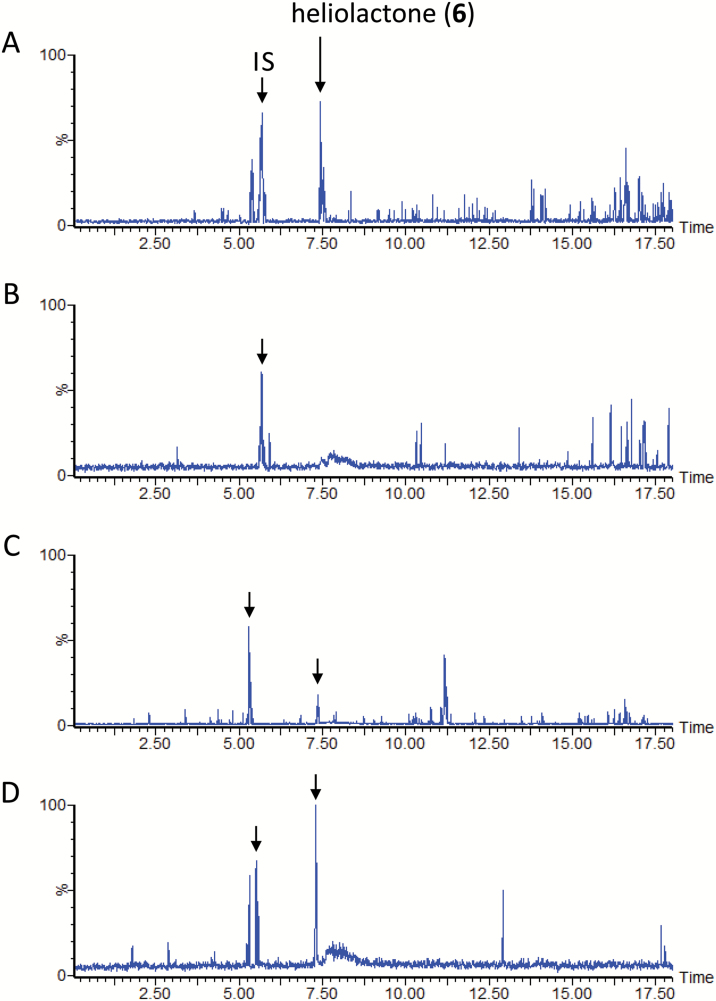
Liquid chromatography–tandem mass spectrometry analysis of sunflower root exudates. The total ion current chromatograms were integrations of multiple reaction monitoring chromatograms with transitions selected for strigol, 5-deoxystrigol (5-DS), carlactone (CL), and heliolactone. (A) Aquaculture filtrate of sunflower grown under phosphate-deficient conditions. (B) Aquaculture filtrate of sunflower grown under phosphate-deficient conditions and supplemented with fluridone. (C) Conversion of CL (**1**) to heliolactone (**6**). (D) Conversion of carlactonoic acid (CLA) (**2**) to heliolactone (**6**). (E) Conversion of methyl carlactonoate (MeCLA) (**3**) to heliolactone (**6**). IS, internal standard.

**Fig. 9.**
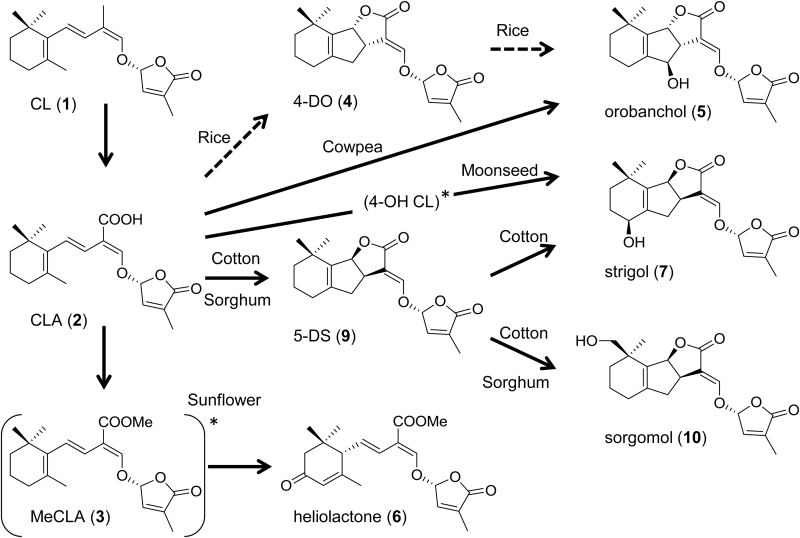
Plant species-dependent biosynthetic pathways for the conversion of carlactone to strigolactones. Solid arrows indicate the conversion steps confirmed in this study. Dashed arrows indicate the established pathways in rice reported by Zhang *et al.* (2014). *Intermediacy of the compound is not confirmed.

We identified strigol (Fig. 1, **7**), a canonical SL, in the root culture filtrates of moonseed (*Menispermum dauricum* DC.) as a germination stimulant of *Striga hermonthica* Del. Benth. (Yasuda *et al.*, 2003), and heliolactone (**6**), a non-canonical SL, from the root exudates of sunflower (*Helianthus annuus* L.), as a germination stimulant of *Orobanche cumana* Wallr. (Ueno *et al.*, 2014). We elucidated the true structures of orobanchol (**5**) and its acetate alectrol (Fig. 1, **8**) produced by cowpea [*Vigna unguiculata* (L.) Walp.] and red clover (*Trifolium pratense* L.) (Ueno *et al.*, 2011, 2015) 10 and 20 years ago, respectively, after their structures were proposed (Müller *et al.*, 1992; Hirayama and Mori, 1999). We also showed the conversion of 5-deoxystrigol (5-DS; Fig. 1, **9**) to sorgomol (Fig. 1, **10**) in hydroponically grown sorghum [*Sorghum bicolor* (L) Moench] (Motonami *et al.*, 2013), which produces both 5-DS (**9**) and sorgomol (Fig. 1, **10**). However, deoxy SL and its oxidized form are not always co-produced. For example, 5-DS (**9**) and 4-DO (**4**) are below the detection limit in moonseed root culture filtrate and aquaculture filtrates of cowpea and red clover, respectively. The present study aimed to obtain more insight into the biosynthetic pathway of oxidized SLs, with special attention to the involvement of 4-DO (**4**) and 5-DS (**9**). Aquacultures of sorghum, cotton, and cowpea, and moonseed root culture were treated with fluridone, a phytoene desaturase inhibitor, to reduce endogenous production of SLs to negligible levels. Subsequently, the plausible biosynthetic SL precursors CL (**1**), CLA (**2**), 4-DO (**4**), and 5-DS (**9**) were exogenously applied. Formation of oxidized SLs from the plausible precursors was analysed using liquid chromatography–tandem mass spectrometry (LC-MS/MS). In a similar manner, sunflower aquaculture was treated with fluridone, and the conversion of plausible biosynthetic precursors CL (**1**) and CLA (**2**) to the non-canonical SL heliolactone (**6**) was investigated.

## Materials and methods

### General

The LC-MS/MS analyses were performed using an LC-MS system (Waters, Milford, MA, USA) consisting of an Acquity ultra-performance liquid chromatograph and an Acquity quadruple tandem mass spectrometer (TQ Detector). The data acquisition and analyses were performed using MassLynx v. 4.1 software (Waters). NMR, circular dichroism (CD), and electron ionization MS spectra were recorded on a JMN-AL 300 spectrometer (JEOL, Tokyo, Japan), J-805 spectropolarimeter (JASCO, Tokyo, Japan), and JMS-700 spectrometer (JEOL), respectively.

### Chemicals

Fluridone was purchased from Wako (Osaka, Japan). CL (**1**) was prepared from 9-*cis*-β-carotene purchased from Carote*Nature* GmbH (Ostermundigen, Switzerland) by using recombinant sorghum CCD7 and CCD8 proteins (SbCCD7 and SbCCD8) as reported by Alder *et al.* (2012) with modifications as described below. CLA (**2**) and MeCLA (**3**) were synthesized according to the method of Abe *et al.* (2014). 4-DO (**4**), strigol (**7**), 5-DS (**9**), 2ʹ-*epi*-strigol, and [6ʹ-D]5-DS were prepared as previously reported (Nomura *et al.*, 2013; Motonami *et al.*, 2013). Strigyl acetate (Fig. 1, **11**) was obtained by the acetylation of strigol (**7**) according to Frischmuth *et al.* (1991).

### Expression of SbCCD7 and SbCCD8 and preparation of carlactone (1)

*Escherichia coli* cells of strain BL21 were transformed with pGEX-SbCCD7 and pGEX-SbCCD8 constructs. Codons unfavorable for *E. coli* were replaced by modifying the nucleotide sequences of cDNAs in order to enrich the A/T residues without changing the amino acid sequence. The transformed cells were grown overnight in Luria–Bertani (LB) medium supplemented with 100 μg ml^−1^ ampicillin and incubated in 250 ml modified LB medium containing the same concentration of ampicillin. The medium, incubated at 37 °C with continuous shaking at 200 rpm for 3 h, was subsequently supplemented with 0.1 mM isopropyl β-D-thiogalactoside. The culture was subsequently incubated at 18 °C with continuous shaking at 150 rpm for 24 h, and *E. coli* cells were harvested by centrifugation at 13 420 *g* for 3 min. The resulting spheroplasts, sonicated in 200 mM HEPES–NaOH (pH 7.8), were subjected to centrifugation at 13 420 *g* for 3 min. The supernatant was subjected to enzyme reactions as described by Alder *et al.* (2012). A reaction product exhibiting identical chromatographic behavior to an authentic sample of CL was purified using semi-preparative HPLC. The CD spectrum showed positive maxima at 218 and 266 nm and matched that reported for 11*R*-carlactone (Seto *et al.*, 2014).

### Plant materials

Root culture of moonseed, a high strigol producer, was established and maintained as described by Yasuda *et al.* (2003). A high sorgomol-producing sorghum cultivar, Sudax, and a high orobanchol-producing cowpea cultivar, B301, were selected as reported previously (Ueno *et al.*, 2011; Motonami *et al.*, 2013). Seeds of sunflower were purchased from Takii Co. Ltd (Kyoto, Japan). Seeds of cotton (*Gossypium arboreum* L. ‘Wawata’ and *Gossypium hirsutum* L. ‘Tonko’) were purchased from Cotton Bank (Mashiko, Japan).

### Feeding of SL precursors

Sorghum seedlings were transferred to test tubes and grown hydroponically in Long Ashton nutrient solution (two-fifths strength) in a growth chamber at 28 °C, with a 16 h light/8 h dark photoperiod. Cotton seedlings were transferred to test tubes and grown hydroponically in Hoagland nutrient solution (one-fourth strength) in a growth chamber under the same temperature and photoperiod conditions as those for the sorghum seedlings. Cowpea and sunflower seedlings were transferred to test tubes and grown hydroponically in Hoagland nutrient solution (one-fourth strength) in a growth chamber at 23 °C and a 16 h light/8 h dark photoperiod. After 2 weeks, the culture medium was replaced by tap water supplemented with 1 µM fluridone, an inhibitor of phytoene desaturase, a key enzyme involved in carotenoid biosynthesis. Three days later, plants were transferred to test tubes containing tap water (50 ml) with any one of the SL precursors CL (**1**) (final concentration, 0.18 µM), CLA (**2**) (0.1 µM), 4-DO (**4**) (0.012 µM), and 5-DS (**9**) (0.012 µM) and incubated for 24 h before filtration. Each aquaculture filtrate was supplemented with *epi*-strigol (50 pmol) as an internal standard and then extracted with EtOAc (15 ml×3). The combined organic layer, washed with 0.2 M K_2_HPO_4_ (pH 8.3) and dried over Na_2_SO_4_, was concentrated *in vacuo*. Each crude EtOAc extract was subjected to column chromatography on silica gel (0.4 g). Strigolactones were eluted with 30% EtOAc in CHCl_3_. Each eluate, dried *in vacuo*, was re-dissolved in 100 μl acetonitrile. For sunflower, owing to low production of heliolactone, three plantlets were transferred to separate plastic cups, each containing 200 ml tap water. Fluoridone (1 µM) was added to each cup and the aquaculture, supplemented with any of the plausible biosynthetic precursors as described above, was incubated for 24 h and filtered. Each filtrate was extracted with EtOAc (50 ml×3), and SLs semi-purified as above were dissolved in 100 μl acetonitrile. An aliquot (5 μl) of each resulting solution was subjected to LC-MS/MS analysis.

Moonseed roots, transferred 50 d after sub-culturing to 25 ml of fresh B5 medium deficient in phosphorus and supplemented with fluridone at 1 μM, were incubated at 26 °C for 24 h before replacement of the medium with fresh phosphorus-deficient B5 medium supplemented with fluridone (1 μM) and CL (**1**), CLA (**2**), or 5-DS (**9**). The cultures were re-incubated for 24 h, and the SLs were extracted, semi-purified, and analysed as described above.

### Expression of AtCYP711A1

*Escherichia coli* cells, strain DH5α, were transformed with a pCW-AtCYP711A1 construct. Codons unfavorable for *E. coli* were replaced by modifying the nucleotide sequence of the cDNA in order to enrich A/T residues without changing the amino acid sequence. The transformed cells, grown overnight in LB medium supplemented with 100 μg ml^−1^ ampicillin, were subsequently incubated in 50 ml modified Terrific Broth medium containing 100 μg ml^−1^ ampicillin, 0.2% w/v glucose, and 0.5 mM γ-aminolevulinic acid. The medium was incubated at 37 °C with continuous shaking at 225 rpm for 2.5 h and subsequently supplemented with isopropyl β-D-thiogalactoside (0.1 mM) and chloramphenicol (1 μg ml^−1^). The culture was incubated at 25 °C with continuous shaking at 150 rpm for 72 h before *E. coli* cells were harvested by centrifugation at 2330×*g* for 20 min. The resulting spheroplasts, sonicated in a buffer solution containing 50 mM potassium phosphate (pH 7.25), 20% (w/v) glycerol, 1 mM EDTA, and 0.1 mM dithiothreitol, were subjected to centrifugation at 100 000 *g* for 1 h. The pellets were sonicated in the same buffer, and the resulting microsomal fractions were subjected to enzyme reactions. The reaction mixture (250 µl), containing 50 mM potassium phosphate buffer (pH 7.25), 30 µM CL, 2.5 mM NADPH, NADPH-cytochrome P450 reductase, and 25 µl microsomal fractions, was incubated at 30 °C for 1 h. The reaction was terminated by adding 25 µl 2 M HCl. The mixture was extracted with EtOAc (250 µl), followed by evaporation of the solvent *in vacuo*. The residue, dissolved in 50 μl acetonitrile, was subjected to LC-MS/MS analysis.

### SL analyses

SL fractions obtained as described above from root exudates were analysed using LC-MS/MS to evaluate the SLs quantitatively and qualitatively. The ultra-performance liquid chromatograph parameters were as follows: column: 100 × 2.0 mm, 2.5 μm, COSMOSIL-packed column 2.5C_18_-MS-II, 30 °C; solvent: 50–100% MeOH in H_2_O (0–20 min, linear gradient); flow rate: 0.2 ml min^−1^. The mass spectrometer was operated in the positive electrospray ionization (ESI) mode for CL, 19-OH-CL, sorgomol, strigol, strigyl acetate, orobanchol, orobanchyl acetate (alectrol), 5-DS, 4-DO, heliolactone, and MeCLA. The capillary voltage was 3 kV, source temperature was 120 °C, and desolvation gas temperature was 350 °C. The nebulizer and desolvation N_2_ gas flow rates were 50 and 600 l h^−1^, respectively. Fragmentation was performed using collision-induced dissociation with argon at 0.1 ml min^−1^. CLA was measured in the negative ESI mode. Multiple reaction monitoring (MRM) was used to detect the presence of precursors and SLs. The MRM transitions, set according to the mass spectra obtained for authentic samples, are summarized in Table 1. *epi*-Strigol, added as an internal standard, was detected under the same conditions as those for strigol. Metabolites derived from CL in moonseed were probed by performing a parent ion scan on the fragment ion at *m*/*z* 97, which corresponds to the D-ring, over the range of 50–400 *m*/*z*, with a cone voltage of 30 V and collision energy of 20 eV. Three products were detected, one of which exhibited major ions at *m*/*z* 319 and *m*/*z* 301.

**Table 1.** Conditions used for the detection of strigolactones with liquid chromatography–tandem mass spectrometry

Strigolactone	Scan mode	Transition (*m*/*z*)	Cone voltage (V)	Collision energy (eV)
CL (**1**)	+	303.1>97.0	30	18
19-OH-CL	+	301.2>185.1	30	18
CLA (**2**)	−	331.2>113.0	30	18
MeCLA (**3**)	+	347.2>97.0	35	20
4-DO (**4**), 5-DS (**9**)	+	331.2>216.1	28	18
	+	331.2>97.0	28	18
Orobanchol (**5**)	+	347.1>233.1	32	14
	+	347.1>205.1	32	14
	+	347.1>97.0	32	14
Heliolactone (**6**)	+	361.2>233.1	35	18
	+	361.2>97.0	35	18
Strigol (**7**), *epi*-strigol (IS)	+	369.1>272.1	38	20
	+	329.1>97.0	30	20
Orobanchyl acetate (alectrol) (**8**)	+	411.1>254.1	42	20
	+	389.2>233.1	40	18
Sorgomol (**10**)	+	364.1>317.1	15	16
	+	317.1>97.0	30	20
Strigyl acetate (**11**)	+	411.2>254.1	45	20
	+	406.1>215.1	20	18

Numbers in bold for the strigolactones refer to Fig. 1. IS, internal standard.

## Results

### SL biosynthesis in sorghum

The LC-MS/MS analysis results confirmed the SL profile previously reported for the aquaculture filtrate of sorghum cultivar Sudax (Motonami *et al.*, 2013). In the culture filtrate, 5-DS (**9**) and sorgomol (**10**) were minor and major SLs, respectively (Fig. 2A). Fluridone restricted the production of both SLs to negligible levels (Fig. 2B). Concurrent application of CL (**1**) to the aquaculture partially restored the accumulation of 5-DS (**9**) and sorgomol (**10**), and the recovery of the exogenously applied CL was below the detection limit (Fig. 2C). In the same experiment, CLA (**2**) was detected, although its signal was weak (Fig. 3A). Peaks of 5-DS (**9**) and sorgomol (**10**) were also detected when CLA (**2**) was applied to the culture (Fig. 2D). The efficient conversion of 5-DS (**9**) to sorgomol (**10**) (Fig. 2E) was consistent with the findings of our previous study (Motonami *et al.*, 2013).

### SL biosynthesis in cotton

Treatment of the cotton cultivars Wawata and Tonko with fluridone reduced SL accumulation to negligible levels (Supplementary Fig. S1B at *JXB* online and Fig. 4B). Feeding CL (**1**), CLA (**2**), and 5-DS (**9**) to fluridone-treated Wawata culture (Supplementary Fig. S1) resulted in the same SL profile as that for Sudax sorghum (Fig. 2). However, unlike in sorghum, feeding the cotton cultivar aquaculture with CL (**1**) led to relatively higher accumulation of CLA (**2**) (Fig. 3B). Conversely, the cotton cultivar Tonko showed a different SL profile from that of Wawata (Fig. 4A). Tonko produces 5-DS (**9**), strigol (**7**), and strigyl acetate (**11**). CL (**1**) was converted to CLA (**2**) in Tonko as efficiently as it was in Wawata (Fig. 3C). Feeding CL (**1**) (Fig. 4C) and CLA (**2**) (Fig. 4D) generated the signals of 5-DS (**9**), strigol (**7**), and strigyl acetate (**11**) with no sorgomol (**10**). Further, 5-DS (**9**) was efficiently converted to strigol (**7**) and strigyl acetate (**11**), but not to sorgomol (**10**) (Fig. 4E).

### SL biosynthesis in moonseed

In the moonseed root culture, a strigol (**7**) producer (Fig. 5A), 5-DS (**9**) was below the detection limit. Treatment of the culture with fluridone significantly decreased the accumulation of strigol (**7**) (Fig. 5B). CL (**1**) was converted to CLA (**2**) (Fig. 3D). The culture converted both CL (**1**) and CLA (**2**) to strigol (**7**) (Fig. 5C, D). 5-DS (**9**) was not detected in either culture. However, a small signal of strigol was detected after the culture was fed with 5-DS (**9**) (Fig. 5E). In a separate experiment, feeding the culture with [6ʹ-D]5-DS confirmed that the labeled substrate with a deuterium atom was not converted to strigol (**6**) (data not shown). Accordingly, the signal of strigol detected in Fig. 5E was not owing to the conversion of exogenously applied 5-DS, but an endogenous product that escaped from the biosynthesis inhibition by fluridone (Fig. 5B).

Feeding moonseed root culture with CL (**1**) at 10-fold concentration, in addition to strigol (retention time (Rt) 7.0 min), resulted in major peaks at Rt 8.3 min, 9.6 min, and 10.1 min (Fig. 6A). The ESI-MS analyses indicated that the unidentified peaks are associated with molecular masses of 316 Da (Fig. 6C), 318 Da (Fig. 6D), and 316 Da (Fig. 6E), respectively. The peak at Rt 9.6 min showed a fragmentation pattern similar to that of strigol (Fig. 6B) and a fragment ion of *m*/*z* 301 (Fig. 6D), corresponding to [M + H − H_2_O], suggesting a hydroxylated product of CL (**1**). Ions corresponding to dehydrated fragments were not observed in compounds eluted at Rt 8.3 min (Fig. 6C) and Rt 10.1 min (Fig. 6E). Abe *et al.* (2014) reported that 19-hydroxy CL was detected along with CLA (**2**) in the enzyme reaction mixture of recombinant AtCYP711A1 protein and CL (**1**) as a substrate. Our experiments confirmed their findings (see Supplementary Fig. S2). The chromatographic behavior of the unidentified product eluted at Rt 9.6 min (Fig. 6A) differed from that of the 19-hydroxy CL eluted at Rt 13.0 min (Supplementary Fig. S2C). The possibility that the product was 18-hydroxy CL cannot be ruled out because its chromatographic behavior has not been reported elsewhere, and no authentic sample is available for comparison. However, considering that strigol (**7**) is the only SL identified in the moonseed culture and has a hydroxy group at C-5 (corresponding to C-4 in the carlactone skeleton), the unidentified metabolite eluted at Rt 9.6 min would probably be 4-hydroxy CL. The fact that 5-DS (**9**) was below the detection limit (Fig. 5A), that exogenously applied 5-DS (**9**) was not converted to strigol (**7**), but metabolized or decomposed (Fig. 5E), and that CL (**1**) might be hydroxylated at C-4 (Fig. 6) are evidence that 5-DS (**9**) is not a biosynthetic precursor of strigol (**7**) in moonseed.

### SL biosynthesis in cowpea

The production of orobanchol (**5**) and its acetate alectrol (**8**) in cowpea aquaculture was confirmed, but 4-DO (**4**) was below the detection limit (Fig. 7A). The plant converted CL (**1**) to CLA (**2**) (Fig. 3E), orobanchol (**5**), and alectrol (**8**) (Fig. 7C). It also converted CLA (**2**) to the SLs (Fig. 7D). Nevertheless, 4-DO (**4**) was not converted to orobanchol (**5**) or alectrol (**8**) (Fig. 7E). These results are similar to those obtained from feeding experiments with moonseed root culture. They also suggest that hydroxylated SLs can be synthesized without having to pass through their deoxy SL as a precursor. However, no possible intermediate between CLA and orobanchol was detected in cowpea.

### SL biosynthesis in sunflower

In sunflower, heliolactone production (Fig. 8A) was inhibited by fluridone (Fig. 8B). CL (**1**) was converted to CLA (**2**) (Fig. 3F) and heliolactone (**6**) (Fig. 8C). In addition, CLA (**2**) was converted to MeCLA (Supplementary Fig. S3D) and heliolactone (**6**) (Fig. 8D).

## Discussion

### The common and species-specific pathways of SL biosynthesis

In all plant cultures tested in this study, SL production was effectively restricted by fluridone, a phytoene desaturase inhibitor, confirming that both canonical and non-canonical SLs are derived from carotenoids. The conversion of CL (**1**) to CLA (**2**) was found to be a common pathway (Fig. 3). CLA (**2**) is converted to canonical or non-canonical SLs in all plants. However, the exact type of SL produced varies across plant species.

Elucidation of the genuine structures of orobanchol and alectrol (Ueno *et al.*, 2015) enabled the classification of canonical SLs into two groups: the strigol-type (**7**, **9**, **10**, **11**) with 3a*R*8b*S* configuration and the orobanchol-type (**4**, **5**, **8**) with 3a*S*8b*R* configuration. Both types of SLs have the C2ʹ*R* configuration in common. According to biogenetic considerations (Rani *et al.*, 2008), allylic hydroxylation converts 4-DO (**4**) and 5-DS (**9**) to orobanchol (**5**) and strigol (**7**), respectively, whereas homoallylic hydroxylation converts 5-DS to sorgomol (**10**). Indeed, 5-DS (**9**) was converted to sorgomol (**10**) in sorghum, confirming the intermediacy of the deoxy SL for the oxygenated SL biosynthesis (Motonami *et al.*, 2013). Given that CLA (**2**) is a precursor of all SLs, the diversity of canonical SLs can be explained by the BC-ring formation mechanism, the subsequent regio- and stereo-selective hydroxylation, and further modifications of the hydroxy group.

Only limited literature is available on BC-ring formation associated with canonical SLs. Zhang *et al.* (2014) reported that 4-DO (**4**) is formed from CLA (**2**) through multistep oxidation reactions catalysed by CLA oxidase. This enzyme is encoded by one of five rice *MAX1* homologs, namely *Os01g0700900*. The authors also reported that 4-DO (**4**) was converted to orobanchol (**5**) by orobanchol synthase, which is encoded by another rice *MAX1* homolog, *Os01g0701400* (Zhang *et al.*, 2014). According to the proposed scheme for the bioconversion of CL into SLs (Zwanenburg *et al.*, 2016), C-18 and C-19 of CL are oxidized to alcohol and carboxylic acid, respectively. Proton transfer from the carboxylic acid to the alcohol provides a species that is prone to undergoing a concerted cyclization to form the BC-ring. The present study revealed that deoxy SL is not always an intermediate in oxygenated SL biosynthesis, and the conversion routes of CLA (**2**) to SLs are different even among plants that produce the same oxygenated SL as revealed in moonseed and the cotton cultivar Tonko, both of which produce strigol (**7**).

### Co-production of deoxy SL and its corresponding oxygenated SL

5-DS has been detected in sorghum (Fig. 2A) and cotton (Fig. 4A), and 4-DO in rice and tobacco (Xie *et al.*, 2013). Nevertheless, deoxy SL and its corresponding oxygenated SL is not always co-produced. Moonseed produces strigol, but only undetectable amounts of 5-DS (Fig. 5A), whereas cowpea produces large amounts of orobanchol and alectrol, but negligible quantities of 4-DO (Fig. 7A). Conversion of CLA (**2**) to 5-DS (**9**) or 5-DS to strigol (**7**) was not detected in the moonseed culture. In cowpea, the conversion of CLA (**2**) to 4-DO (**4**) and 4-DO to orobanchol (**5**) was not found. In plant species in which deoxy SLs did not participate in oxygenated SL biosynthesis, the recovery of exogenously applied deoxy SLs was very poor. No exogenously applied 5-DS (**9**) was detected in moonseed (Fig. 5E), and only a limited amount of 4-DO (**4**) was found in cowpea (Fig. 7E). Their conversion to strigol (**7**) or orobanchol (**5**) was not detected. Conversely, significant amounts of unconverted 5-DS (**9**) accumulated in sorghum and in both cotton cultivars (Figs 2E and 4E, Supplementary Fig. S1E). These plants converted the substrate to strigol (**7**) or sorgomol (**10**). The results suggest that SLs irrelevant to endogenous SL biosynthesis are recognized and treated by plants as xenobiotics, which might be decomposed, converted to extraction-recalcitrant forms, or retained in the plants. Thus, co-production is an indication that deoxy SLs are converted to their corresponding oxygenated SLs in plants.

### Strigol biosynthesis in moonseed and orobanchol biosynthesis in cowpea

The conversions of CL (**1**) and CLA (**2**) to strigol (**7**) (Fig. 5C, D) as well as that of CL to CLA (Fig. 3D) and putative 4-hydroxy CL (Fig. 6) suggest that a metabolic grid exists in moonseed that uses 4-hydroxy CLA as an immediate precursor of strigol (**7**). Excess amounts of CL and/or its hydroxylated product may be recognized as xenobiotics and subsequently catabolized to yield the products eluting at Rt 8.3 min and Rt 10.1 min (Fig. 6A). The irrelevance of 5-DS in strigol biosynthesis in moonseed is rational if hydroxylation at C-4 precedes BC-ring formation. However, no possible intermediate was detected between CLA (**2**) and orobanchol (**5**) in cowpea root exudates. Notably, gold-catalysed cyclization of *cis*-2,4-dien-1-als generates the corresponding cyclic compounds (Lin *et al.*, 2007). If the C-18 of CLA is oxidized in two steps to form an aldehyde, and then the aldehyde is subjected to a similar cyclization procedure via a catalyst, orobanchol isomers could be generated. This reaction mechanism rationalizes how CLA (**2**) is converted to orobanchol (**5**) without passing through 4-DO (**4**), although whether elaborate machinery exists, such as a dirigent protein enabling stereo-controlled BC-ring closure, needs to be determined.

### Heliolactone biosynthesis in sunflower

The bioconversion of CL (**1**) and CLA (**2**) to heliolactone (**6**) was unambiguously demonstrated (Fig. 8). Heliolactone (**6**) is a non-canonical SL having a hydroxylated ε-ring, whereas CL (**1**) and CLA (**2**) have the same β-ring as β-carotene. Accordingly, the conversion of CL (**1**) to heliolactone (**6**) requires double bond migration and hydroxylation at C-3, followed by oxidation to ketone in the A-ring. However, the participation of precursors such as α-carotene, lutein, and zeaxanthin derived from other plant carotenoids, cannot be ruled out. These carotenoids have a β-ring and an ε-ring, a hydroxylated ε-ring and a hydroxylated β-ring, and hydroxylated β-rings, respectively. The isomerization of α-carotene to its 9-*cis* isomer by rice D27 (Bruno and Al-Babili, 2016) and the oxidative cleavage of 9-*cis*-lutein and 9-*cis*-zeaxanthin by Arabidopsis and pea CCD7 (Bruno *et al.*, 2014) support the involvement of these carotenoids in heliolactone biosynthesis. A previous study (Ueno *et al.*, 2014) could not provide the absolute configuration at C-11 of heliolactone (**6**) because the Cotton effect around 250 nm, which indicates the configuration at C-2ʹ of SLs (Welzel *et al.* 1999), was hidden by the superimposition of the curve derived from the cyclohexenone/dienoate chromophoric pair. CL (**1**) used in this experiment was prepared by incubating 9-*cis*-β-carotene with an enzyme mixture consisting of SbCCD7 and SbCCD8. The CD spectrum of the enzymatically prepared CL indicated its C11*R* configuration, the same as that prepared by OsCCD8 (Zhang *et al.*, 2014). Accordingly, the conversion of CL (**1**) to heliolactone (**6**) (Fig. 8C) provides evidence for the C11*R* configuration of the latter molecule.

The results obtained in this study are summarized in Fig. 9. In conclusion, the conversion of CL (**1**) to CLA (**2**) was a reaction common to all plants investigated irrespective of whether they produced canonical or non-canonical SLs. 5-DS (**9**) was not a precursor of strigol (**7**) in moonseed, and 4-DO (**4**) was not a precursor of orobanchol (**5**) in cowpea. These results indicate that deoxy SLs are not necessarily the precursors of hydroxy SLs, although the conversion of 5-DS (**9**) to sorgomol (**10**) and 4-DO (**4**) to orobanchol (**5**) was established in sorghum (Motonami *et al.*, 2013) and rice (Zhang *et al.*, 2014), respectively. The involvement of deoxy SLs in the biosynthesis of oxygenated SLs is consistent with the detection of deoxy SLs in root exudates. Elucidation of BC-ring formation mechanisms in plants other than rice might reveal the entire canonical SL biosynthesis pathway. The conversion of CLA (**2**) to the non-canonical SLs MeCLA (**3**) and heliolactone (**6**) was detected in sunflower. Further studies are warranted to comprehensively analyse the biosynthetic pathways of canonical and non-canonical SLs.

## Supplementary data

Supplementary data are available at *JXB* online.

Fig. S1. Conversion of CL to CLA and 19-hydroxy CL by AtCYP711A1.

Fig. S2. Conversion of CL, CLA, and 5-DS to sorgomol in Wawata cotton.

Fig. S3. Conversion of CLA to MeCLA in sunflower.

Supplementary Figures S1-S3
