# Supplementary material for: Evidence for species-dependent biosynthetic pathways for converting carlactone to strigolactones in plants
Source: J Exp Bot. 2017 Dec 23;69(9):2305–18. doi: 10.1093/jxb/erx428 (PMC5913628; doi:10.1093/jxb/erx428)
Supplement: Supplementary Figures S1-S3 [file erx428_suppl_supplementary_figures_s1-s3.pdf]

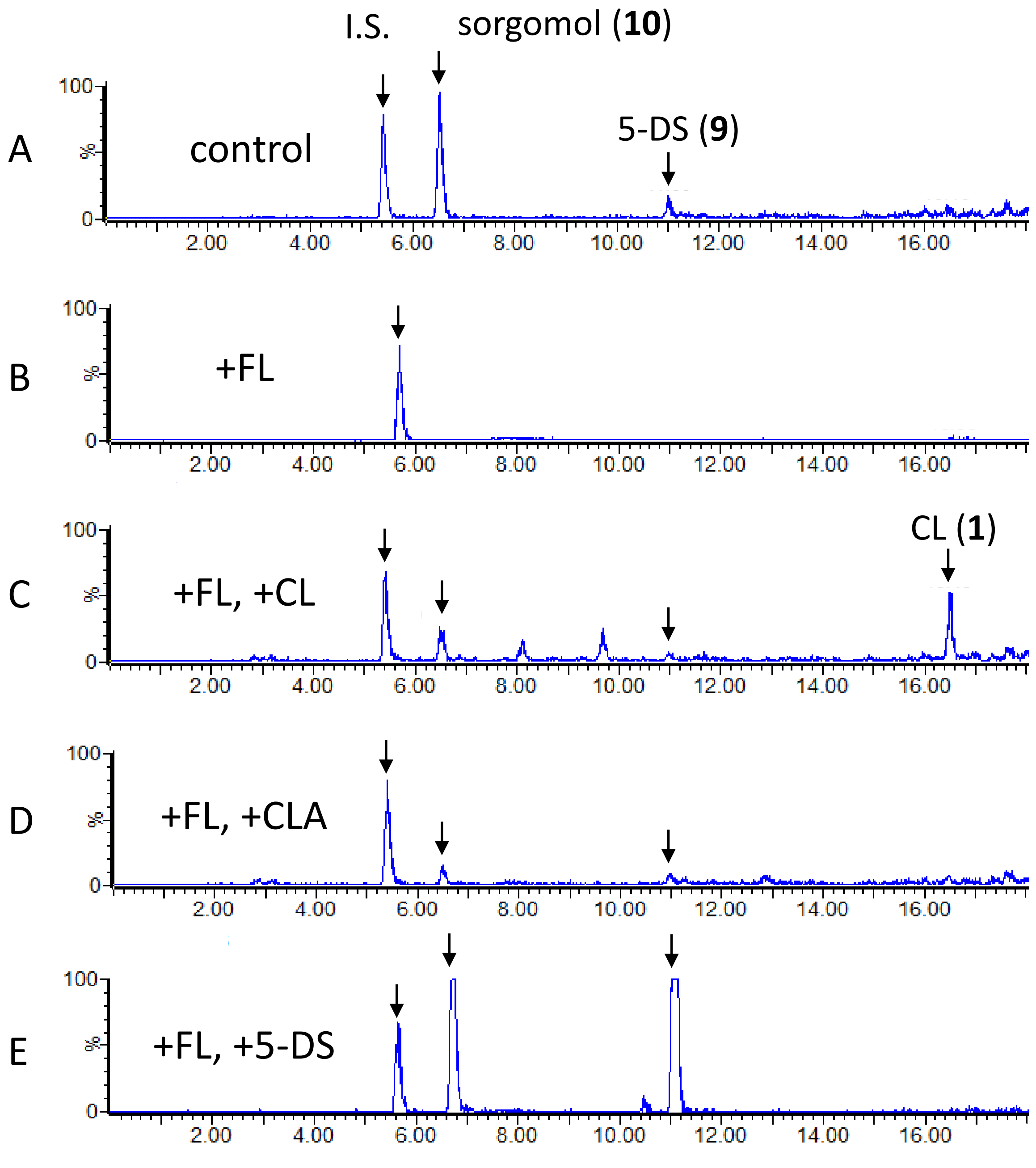

Conversion of CL, CLA, and 5-DS to sorgomol by Wawata cotton. (A) Aquaculture filtrate of Wawata under a phosphate-deficient condition. (B) Aquaculture filtrate of Wawata grown under a phosphate-deficient condition and supplemented with fluridone. (C) Conversion of CL (**1**) to 5-DS (**9**) and sorgomol (**10**). (D) Conversion of carlactonoic acid (CLA) (**2**) to 5-DS (**9**) and sorgomol (**10**). (E) Conversion of 5-DS (**9**) to sorgomol (**10**).

Supplementary Fig. S1

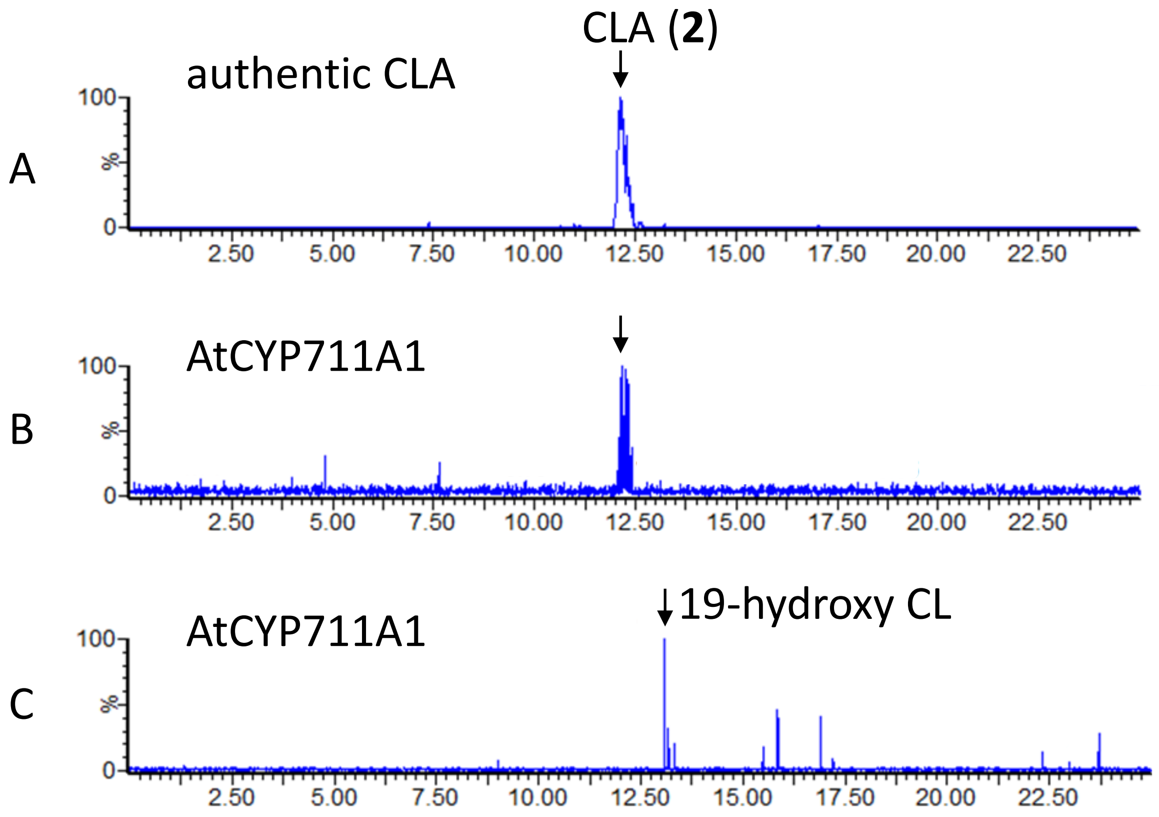

Conversion of CL to CLA and 19-hydroxy CL by AtCYP711A1. (A) Authentic sample of CLA. (B, C) Enzyme reaction mixture of recombinant AtCYP711A protein and CL as a substrate.

Supplementary Fig. S2

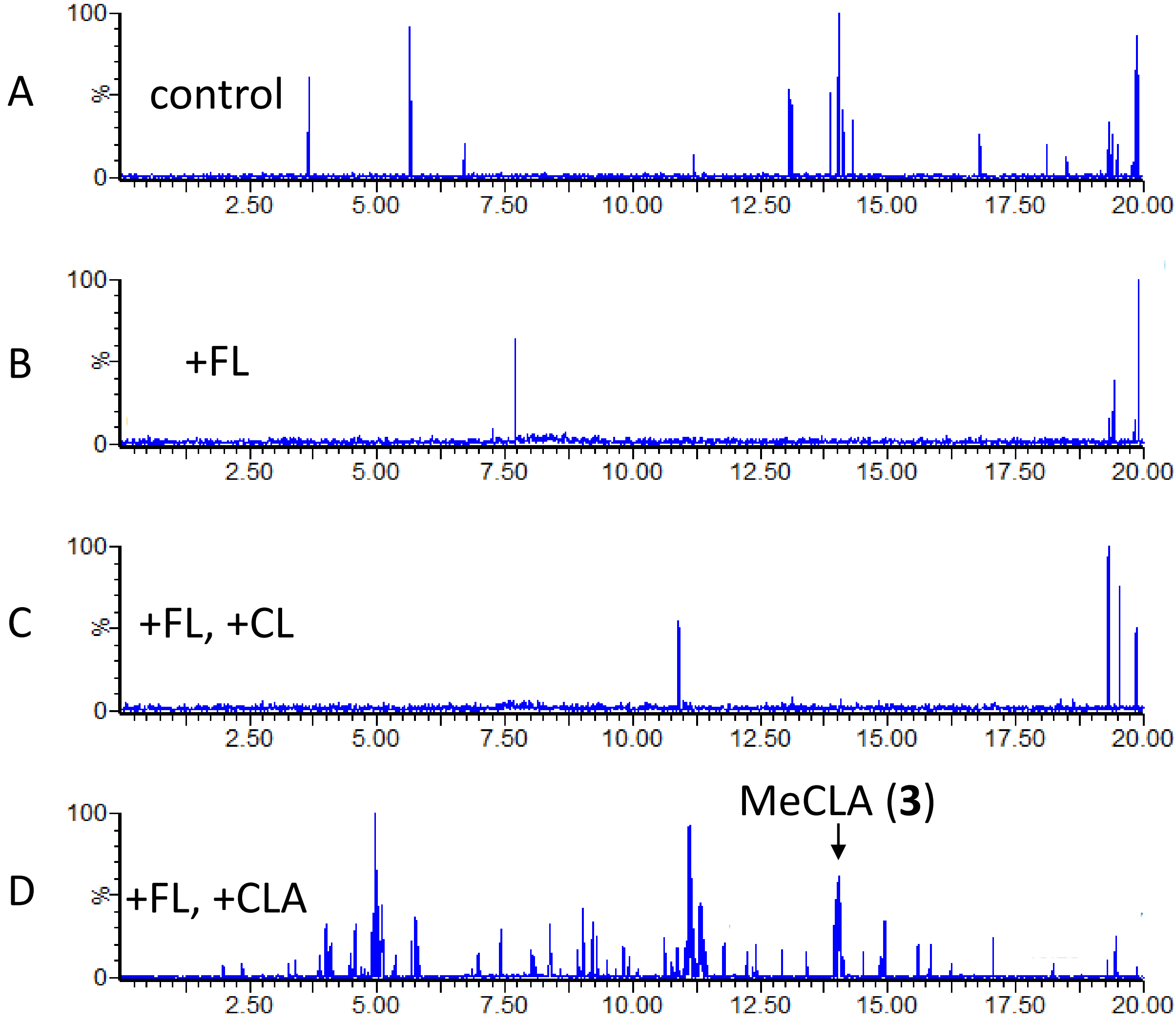

Conversion of CLA to MeCLA by sunflower. Samples in A to D are the same as those in Figure 7. (D) Conversion of carlactonoic acid (CLA) (**2**) to methyl carlactonoate (**3**) was detected.

Supplementary Fig. S3
